# Methods for Baiting and Enriching Fungus-Feeding (Mycophagous) Rhizosphere Bacteria

**DOI:** 10.3389/fmicb.2015.01416

**Published:** 2015-12-22

**Authors:** Max-Bernhard Ballhausen, Johannes A. van Veen, Maria P. J. Hundscheid, Wietse de Boer

**Affiliations:** ^1^Department of Microbial Ecology, Netherlands Institute of EcologyWageningen, Netherlands; ^2^Department of Plant Health, Institute for Vegetable and Ornamental CropsGroßbeeren, Germany; ^3^Institute of Biology Leiden, Leiden UniversityLeiden, Netherlands; ^4^Department of Soil Quality, Wageningen UniversityWageningen, Netherlands

**Keywords:** mycophagy, fungus-feeding, cultivable bacteria, antifungal, rhizosphere, isolation method, phytagel

## Abstract

Mycophagous soil bacteria are able to obtain nutrients from living fungal hyphae. However, with exception of the soil bacterial genus *Collimonas*, occurrence of this feeding strategy has not been well examined. Evaluation of the importance of mycophagy in soil bacterial communities requires targeted isolation methods. In this study, we compared two different approaches to obtain mycophagous bacteria from rhizospheric soil. A short-term method based on baiting for bacteria that can rapidly adhere to fungal hyphae and a long-term method based on the enrichment of bacteria on fungal hyphae via repeated transfer. Hyphae-adhering bacteria were isolated, identified by 16S rDNA sequencing and tested for antifungal activity and the ability to feed on fungi as the sole source of carbon. Both methods yielded a range of potentially mycophagous bacterial isolates with little phylogenetic overlap. We also found indications for feeding preferences among the potentially mycophagous bacteria. Our results indicate that mycophagy could be an important growth strategy for rhizosphere bacteria. To our surprise, we found several potential plant pathogenic bacteria among the mycophagous isolates. We discuss the possible benefits that these bacteria might gain from colonizing fungal hyphae.

## Introduction

Bacteria and fungi commonly co-occur in a variety of habitats (Frey-Klett et al., 2011). The habitat with the highest diversity of both groups is the soil. Here, available nutrients are scarce and thus microorganisms have to compete for them. Soil bacteria have developed different strategies to cope with limited resources, such as the production of toxic secondary metabolites that suppress competitors or the adaptation to specific energy resources (Hibbing et al., 2010). A strategy that has been termed mycophagy is used by soil bacteria of the genus *Collimonas.* These bacteria are known for their ability to exploit living fungi to obtain energy for growth (Leveau and Preston, 2008). *Collimonas* bacteria appear to use a combination of antibiotics and enzymes to get access to organic nutrients present in living fungal hyphae (Leveau et al., 2010). They are especially abundant in (semi-)natural, acidic grassland soils where they can have an impact on the composition of fungal communities, probably due to selective feeding on particular fungal species (Hoppener-Ogawa et al., 2007, 2009). Besides this information, very little is known on the importance of bacterial mycophagy in soil ecosystems. It has, however, been found that bacteria are frequently associated with hyphae of a wide range of fungi, covering all important functional fungal groups. This hints toward the possibility that mycophagous feeding by bacteria might be much more common than currently appreciated (Leveau and Preston, 2008; Nazir et al., 2010; Frey-Klett et al., 2011). In a recent study, Rudnick et al. (2015) could show that rhizosphere fungi are colonized by a diverse group of potentially mycophagous bacteria. Based on these results the authors proposed that fungi (saprotrophic as well as mycorrhizal) take up a substantial part of root-derived carbon, thereby sustaining a mycophagous rhizosphere bacterial community. In this scenario, part of the rhizosphere bacteria would feed as secondary consumers on root derived carbon.

Other aspects that warrant further study on the occurrence of bacterial mycophagy among soil bacterial species are the perspectives to use this feeding strategy for biocontrol of soil-borne pathogenic fungi or for the discovery of novel fungicides (Kamilova et al., 2007; Mela et al., 2011; Fritsche et al., 2014).

Providing proof for mycophagous abilities of a bacterium requires the demonstration that intact living fungi can constitute the only source of nutrients sustaining the growth of the bacterium. In an earlier study, mycophagous growth of *Collimonas* bacteria was demonstrated in microcosms where fungal hyphae that were invading pure sand formed the only source of nutrients (De Boer et al., 2001). This sand microcosm approach was, however, laborious and time consuming, and therefore not suitable to screen a high number of bacterial isolates. We recently developed an improved microcosm system where Phytagel, a very pure agar substitute, was used as the nutrient-poor environment in which growing fungal hyphae encounter bacteria (Rudnick et al., 2015). The Phytagel-assay was used to screen the mycophagous potential of rhizosphere bacterial isolates that were rapidly adhering (24 h) to fungal hyphae. Samples were taken in a nutrient-poor liquid environment (“short-term liquid hyphal baiting”) that was inoculated with bacteria extracted from the rhizosphere of a grass and a sedge. This rapid baiting method combined with the Phytagel-mycophagy assay revealed that the potential to feed on common saprotrophic rhizosphere fungi is taxonomically widespread among rhizosphere bacteria. Besides *Collimonas*-related β*-Proteobacteria*, the community of hyphae colonizing rhizosphere bacteria also comprised α*-* and γ*-Proteobacteria, Actinobacteria*, and *Bacteroidetes*.

This short-term baiting method might, however, bias the recovery of mycophagous bacteria to quickly attaching ones. In order to further explore the mycophagous potential among rhizosphere bacteria and to understand rhizosphere soil as a reservoir of mycophagous bacteria in more detail, the current study introduces a long term baiting “transfer-enrichment” method. Our aim was to determine whether long term baiting, based on repeated transfer of hyphae-adhering bacteria, would yield other mycophagous soil bacteria than the already established short-time baiting method. We show that the two methods yield different groups of rhizosphere bacteria with the ability to feed on saprotrophic as well as phytopathogenic fungi.

## Materials and Methods

### Soil Inocula and Host Fungi

We used filtered soil suspensions from the rhizosphere of sand sedge (*Carex arenaria*) and fescue grass (*Festuca rubra*) to inoculate both microcosm systems. These plant species co-occurred on an inland river dune in the Netherlands, characterized by nutrient-poor sandy soil with low organic matter content. Since the same soil was already used in a previous study, we refer to Rudnick et al. (2015) for a detailed description of the sampling location and of the soil inoculum preparation. In short, rhizosphere soil was suspended in a diluted salt solution, shaken, sonicated and filtered repeatedly, until the filtrate mainly consisted of bacteria (plating on PDA showed no fungal colonies). The plant-pathogen *Rhizoctonia solani* and the saprotrophs *Mucor hiemalis* and *Trichoderma harzianum* were used as host fungi. Details on the origin of the last two fungi are given in Rudnick et al. (2015). *R. solani* (AG22IIIB) was supplied by the Institute of Sugar Beet Research (IRS, The Netherlands).

The two saprotrophic host fungi were selected because they are common rhizosphere fungi for both *C. arenaria* and *F. rubra* (Prenafeta Boldú et al., 2014). The plant pathogenic fungus *R. solani* is a well-known pathogen of grasses (Kohli, 1966; Amaradasa et al., 2013). Before performing the experiments, all fungi were pre-cultured on Potato Dextrose Agar (9.75 gL^-1^ potato dextrose agar; 3.75 gL^-1^ agar) supplemented with the bactericidal antibiotics oxy-tetracycline (Sigma–Aldrich, Zwijndrecht, The Netherlands; 50 mgL^-1^) and streptomycin (Sigma–Aldrich, Zwijndrecht, The Netherlands; 100 mgL^-1^) and subsequently controlled and found free of bacterial contamination by DNA isolation and PCR.

### Short-Term “Liquid Hyphal Baiting” Method

This system was already described in detail in Rudnick et al. (2015) and will be further referred to as “liquid hyphal baiting”. Briefly, *R. solani* was inoculated in a two-compartment Petri-dish in M-medium, containing 1% glucose (Bécard and Fortin, 1988). This medium was solidified with Phytagel (Sigma–Aldrich, St. Louis, MO, USA). Phytagel is an agar substitute composed of interlinked D-sugars, solidified with MgSO_4_. The availability of Phytagel-derived carbon resources for microbial growth is extremely low (Sutherland and Kennedy, 1996). There are, however, a few bacteria that can cleave and metabolize those sugars, probably by using specific enzymes, called gellan lyases (Kennedy and Sutherland, 1994; Stott et al., 2008). During growth, fungi crossed the plastic barrier that separated the two compartments and colonized the second compartment that contained the same medium but without degradable carbon resources. Finally, a plug was cut out of the Phytagel in the second compartment and the slot was filled with liquid medium of the same composition (i.e., free of carbon sources) but without Phytagel as the gelling agent. The fungus was left to colonize the liquid medium (the slot) for 11–13 days at 20°C and subsequently inoculated with the filtered rhizosphere bacterial suspension. Hyphae-adhering bacteria were sampled after 24 h by collecting hyphae with a sterile inoculation loop. Hyphal fragments were subsequently washed in MES (Morpholineethanesulfonic acid) buffer (pH 5.5), containing 1 gL^-1^ KH_2_PO_4_ and 1 gL^-1^ (NH_4_)_2_SO_4,_ to select for only firmly attaching bacteria and processed for bacterial culturing and DNA isolation. In the current article, we present the results of short-term liquid baiting for the plant-pathogenic fungus *R. solani*, whereas results for the saprotrophic fungi *M. hiemalis* and *T. harzianum* have already been described in Rudnick et al. (2015).

### Long-Term Hyphal-Baiting Method (“Transfer-Enrichment”)

**FIGURE 1:**
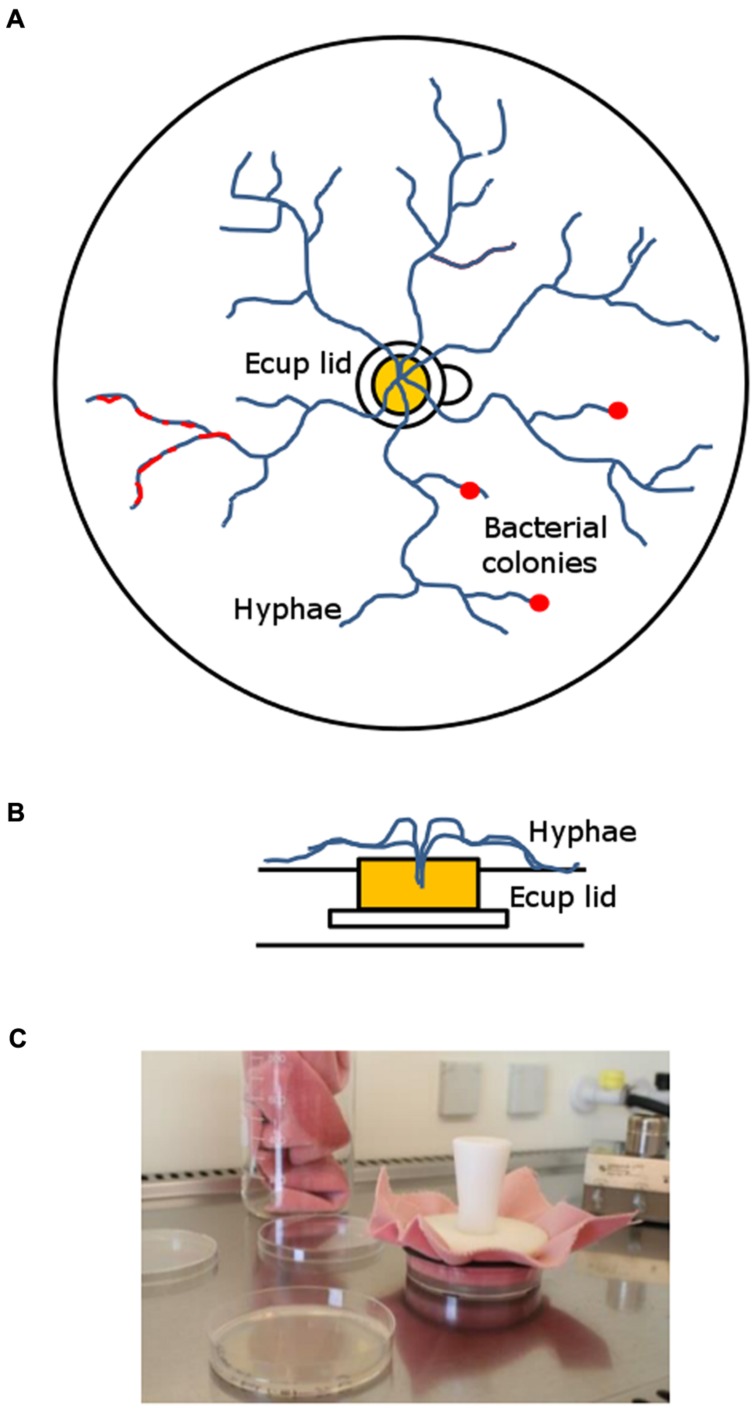
**Long-term hyphal-baiting (“transfer-enrichment”) approach.** **(A)** Top-view and **(B)** side-view. The fungus was inoculated in the lid of an Eppendorf Cup containing Malt extract agar. It overgrew the plastic rim which separated the nutrient containing compartment (Malt extract agar) in the lid from the nutrient-poor Phytagel in the Petri dish. Subsequently, the microcosm was colonized by the fungal hyphae. Bacteria were distributed over the Phytagel compartment after colonization by the hyphae and strains that became enriched appeared as a biofilm or small colonies along fungal hyphae (red). **(C)** Regular transfers were done to promote the enrichment of those bacteria that can exploit fungal hyphae for carbon.

The microcosm system that was developed for the long-term enrichment of mycophagous bacteria consisted of a Petri dish (94 mm dia, 16 mm height), filled with ∼15 ml of 4% wV^-1^ Phytagel (supplemented with 0.74 gL^-1^ MgSO_4_ to solidify). In the middle of the microcosm a sterile lid of an Eppendorf cup was placed and filled with ∼200 μl hot Malt Extract Agar (MEA; 15 gL^-1^ agar, 3 gL^-1^ peptone and 20 gL^-1^ malt extract). After solidification of MEA, fungal cultures were inoculated by introducing small plugs (∼2–3 mm^2^) from fungal MEA pre-cultures. MEA provides a nutrient source for the fungus from which it further colonizes the surrounding nutrient free Phytagel medium. The rim of the Eppendorf cup lid can easily be overgrown by fungal hyphae but prevents diffusion of nutrients into the Phytagel. Hence, carbon derived from fungal hyphae forms the only source of nutrients for bacteria on the Phytagel (See **Figure 1**).

Microcosms were left to be colonized by the three different fungi. Colonization speed differed between fungi: *M. hiemalis* colonized the microcosm in 10–14 days, *T. harzianum* and *R. solani* in 5–7 days. After fungal colonization was completed, microcosms were inoculated with filtered rhizosphere bacterial suspensions (see above). A total amount of 400 μl suspension was carefully distributed over the whole plate, using a pipette. Inoculation was done in a full factorial design, using three replicates. After 14 days of incubation, the developing bacteria were transferred by stamping to a new microcosm that had already been colonized by the same host fungus (see above). Stamping refers to the technique of replica plating (Lederberg and Lederberg, 1952) which has originally been developed to screen for antibiotic resistant bacteria. We used a sterile velvet cloth for consecutive transfer of bacteria that were associated with a fungal species (see **Figure 1C**). This enrichment procedure was repeated every 14 days for six times. Finally, the enriched hyphae-adhering colonies were washed of the Petri dishes, using 2 ml of MES buffer (pH 5.5). We refer to this isolation approach as “transfer-enrichment” throughout the rest of the article.

### Bacterial Isolation, Sequencing, Confrontation- and Mycophagy Assay

A detailed description of isolation and sequencing can be found in Rudnick et al. (2015). Briefly, hyphal fragments collected from both systems were dilution plated on Tryptic Soy Agar (TSA; KH_2_PO_4_ 0.5 gL^-1^, NaCl 2.5 gL^-1^, Yeast extract 0.05 gL^-1^, Tryptone 1.5 gL^-1^ and Agar 10 gL^-1^, pH 6.8) containing fungicides (100 mgL^-1^ cycloheximide (Sigma–Aldrich, Zwijndrecht, The Netherlands) and 50 mgL^-1^ delvocid (DSM, Heanor, UK). Bacterial colonies were randomly picked from different dilutions, ranging from 1:10 to 1:1000 and transferred to fresh TSA plates until they were free of bacterial or fungal contaminants. Since it has been indicated that antifungal activity is an important factor for mycophagous growth of collimonads (Leveau et al., 2010; Mela et al., 2011), isolated bacteria were subsequently scored for the ability to inhibit their host fungus in a confrontation assay. The assay was conducted on Water Yeast Agar (WYA; KH_2_PO_4_ 1 gL^-1^, NaCl 5 gL^-1^, Yeast extract 0.05 gL^-1^ and Agar 20 gL^-1^, pH 6.8) using standard size Petri dishes. This medium has been used to simulate the carbon-limited conditions for microbial growth in soils (De Boer et al., 2007). Bacteria were pre-cultured on TSA and inoculated on a 0.5 cm × 4 cm zone, 4 days before the fungi were introduced as a plug from the margin of an actively growing colony (**Supplementary Figure S1**). Assays were conducted at 20°C and scored after 7 days. Bacteria that were able to stop the growth of the fungus by the creation of a fungus-free inhibition zone in front of the bacterial inoculation patch were scored as antifungal.

The identities of a subset of the inhibitory bacteria were determined by PCR amplification (primers 27f and 1492r (Weisburg et al., 1991)) and Sanger sequencing of the small ribosomal subunit gene (16S rDNA). For PCR chemistry and cycling parameters we refer to Rudnick et al. (2015). Sequences were submitted to the European Nucleotide Archive (ENA). They can be accessed under the accession numbers LM652338–LM652373. Bacterial isolate identification was based on the closest match with the 16S rDNA sequences of cultured strains or environmental sequences of uncultured bacteria in the RDP (Ribosomal database project) database. (https://rdp.cme.msu.edu/). Partial 16S rDNA sequences were aligned and manually curated. Phylogenetic trees were constructed using the Neighbor Joining method with standard settings in MEGA 6 (Tamura et al., 2011). Missing nucleotide data were treated as a complete deletion in the alignment and trees were tested with 100 bootstraps. Other graphs, tables and statistics (*t*-tests) were done in Excel (Microsoft Corp.).

### Mycophagy Assay

Mycophagous abilities of the inhibitory isolates were scored via the mycophagy assay as described in Rudnick et al. (2015). In case of identical sequences, representative isolates were chosen. Briefly, bacteria were inoculated on a Petri-dish, containing Phytagel medium. Subsequently, the host fungus was introduced on a nutrient-rich patch in the middle of a Petri-dish. A metal disc separated the nutrient patch from the content of the Petri-dish, thus preventing diffusion of nutrients into the Phytagel. Natural fungal colony expansion forced the encounter of potentially mycophagous bacteria and the fungal host. After incubation, bacterial cells were washed of the Petri-dish, optical density at 600 nm wavelength (OD_600_) was measured and mycophagy ratios were calculated (OD_600_ treatment/OD_600_ control). We performed a “fungus only” and a “bacteria only” control to account for possible OD increases by hyphal fragments and background bacterial growth (on Phytagel only), respectively, and chose the higher OD_600_ value of the two as a control for the calculation. All measurements were done in triplicates.

### Validation of Mycophagous Growth

We validated that the observed bacterial growth is based on active exploitation of the fungi, rather than passive consumption of compounds exuded by the fungi. For this assay, we used *M. hiemalis* since this was the fungus for which most potential mycophagous isolates were obtained. A plug of *M. hiemalis* was transferred from a nutrient-rich (PDA) plate onto Phytagel as described for the mycophagy assay. After the Phytagel was completely colonized by the fungus, 5 ml MES buffer was added and the microcosm was incubated for 30 min. The buffer was removed with a sterile pipette and different bacterial strains (highest similarity with *Pseudomonas protegens* PGNR1, beta proteobacterium A35-1 (*Burkholderia*), uncultured eubacterium WD202 (*Burkholderia*), *Dyella* sp. ICB487, *Burkholderia* Y86, and *Neisseriaceae* bacterium IGB-41, respectively) were diluted and re-suspended to an OD_600_ of 0.01 in the collected buffer. Bacterial liquid cultures were incubated at 20°C on a horizontal shaker. After 7 days of incubation OD_600_ was measured. Data were checked for homoscedasticity with an *f* test and subsequently triplicate averages of controls (incubation in MES buffer collected from Phytagel plates without fungi) versus treatments (incubation in MES buffer collected from Phytagel plates colonized by *M. hiemalis*) were compared with a two-tailed *t*-test.

## Results

### Hyphae-Adhering Bacteria with Antifungal Properties

Like the short-term “liquid hyphal baiting” method, the long-term “transfer-enrichment” method was successfully used to obtain a phylogenetically diverse group of antifungal bacteria (based on the confrontation assay). With the “liquid hyphal-baiting” method, we retrieved 78 isolates of bacteria adhering to hyphae of *R. solani.* Of these isolates 51 (65%) showed *in vitro* inhibitory activity against the host fungus on WYA. Results of “liquid hyphal baiting” for the other two fungi were already published: 65% of the isolates (*n* = 132) obtained for *M. hiemalis* and 35% of the isolates (*n* = 71) obtained from *T. harzianum* were inhibitory against the respective host fungi (Rudnick et al., 2015). The “transfer-enrichment” method yielded 540 isolates in total (180 from each host fungus), of which 43 (24%), 58 (32%), and 47 (26%) isolates were able to inhibit the growth of their respective host fungus (*M. hiemalis, T. harzianum, R. solani*). In a few cases bacterial isolates obtained by both methods could be assigned to the same species (*P. protegens*) or the same genus (*Burkholderia, Pantoea*, and *Pseudomonas*).

**FIGURE 2:**
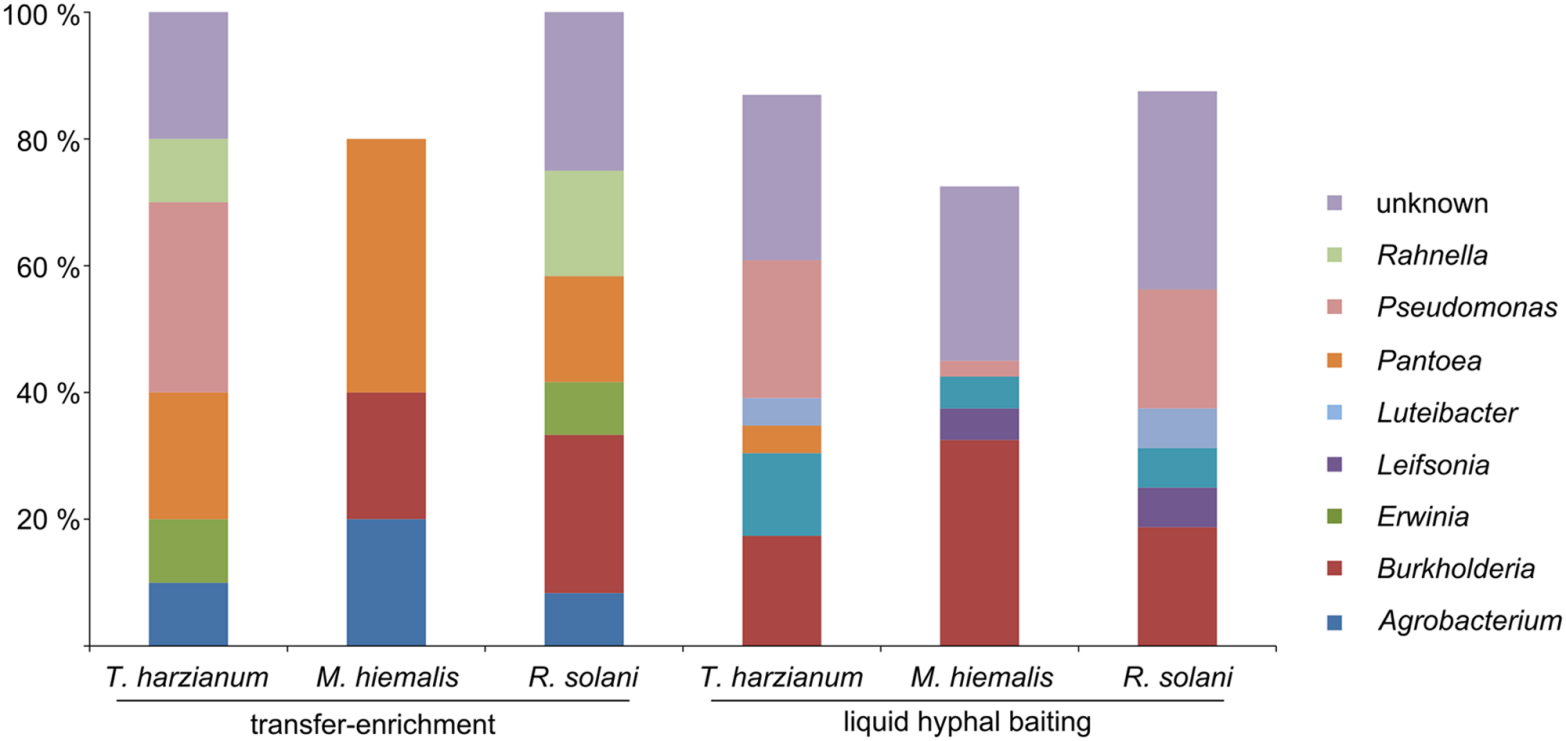
**Relative amount of antifungal, hyphae-adhering bacterial isolates grouped by bacterial genus, the three host fungi (*T. harzianum, M. hiemalis, R. solani*) and the two isolation methods (long-term baiting “transfer-enrichment” and short-term “liquid hyphal-baiting”.** Only genera are displayed of which isolates were obtained that colonized more than one fungal species or that were isolated with both methods.

**FIGURE 3:**
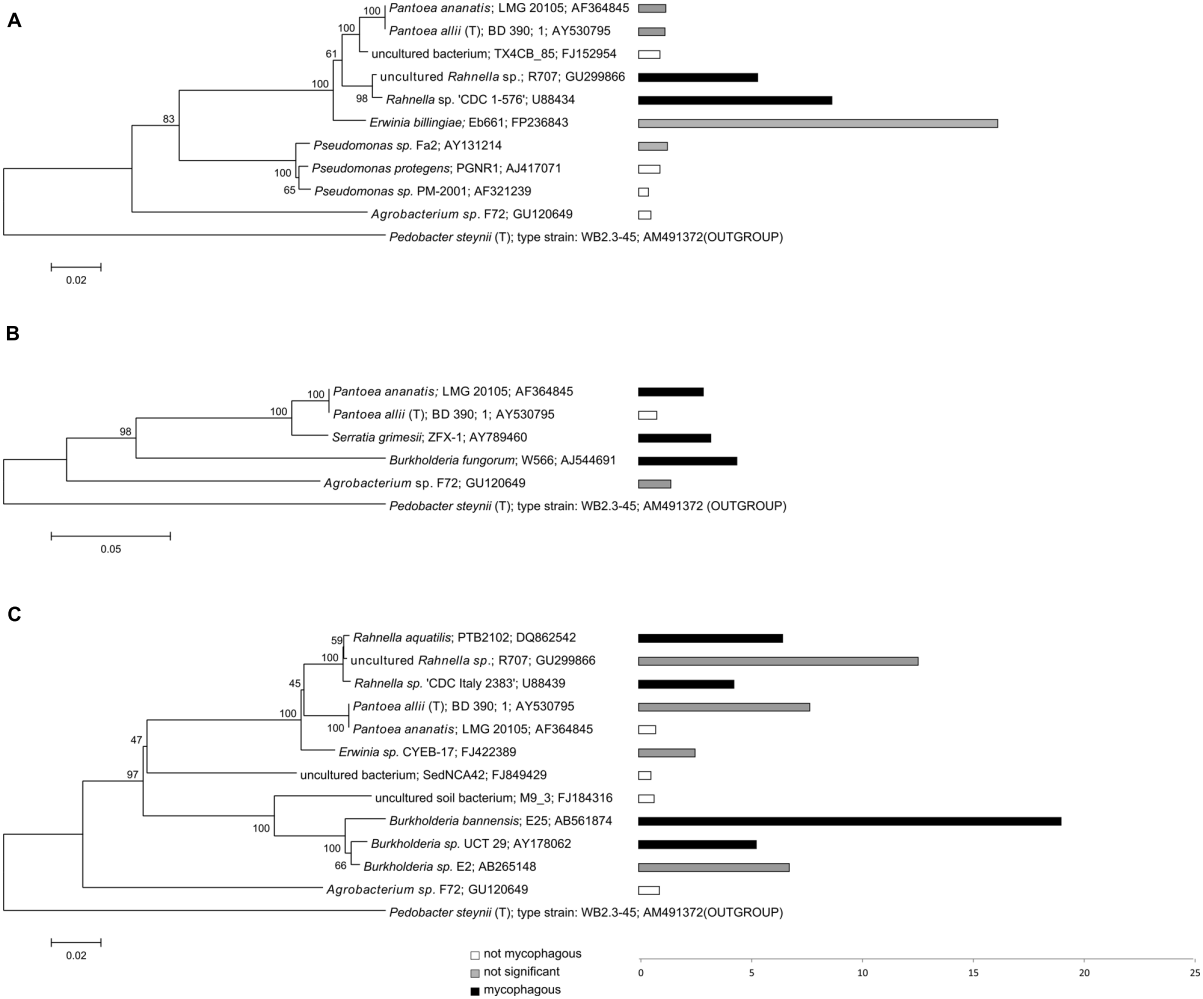
**Phylogeny and potential mycophagous ability of antifungal, hyphae-adhering bacteria retrieved with the “transfer-enrichment” approach.** The indicated bacterial strains are cultured or uncultured bacteria that have the closest match to one or several of the antifungal, hyphae-adhering bacterial isolates. Separate trees are presented for bacteria associated with **(A)** *T. harzianum*, **(B)** *M. hiemalis* and **(C)** *R. solani*. Bars indicate measured mycophagy ratios: black bars representing bacteria demonstrating significant mycophagous growth (ratio > 1; *P* < 0.05), gray bars representing bacteria with possible mycophagous growth (ratio > 1; *P* > 0.05), and white bars represent bacteria with no mycophagous growth (ratio ≤ 1).

Both applied methods to obtain fungus-associated bacteria had a strong effect on the composition of the antifungal bacteria. Strains assigned to the genera *Agrobacterium, Erwinia*, and *Rahnella* were only isolated with the “transfer-enrichment”, whereas strains assigned to the genera *Luteibacter, Leifsonia*, and *Pedobacter* were only obtained with the “liquid hyphal-baiting” method. Bacteria assigned to the genera *Rahnella, Pseudomonas, Pedobacter, Pantoea, Luteibacter, Leifsonia, Erwinia, Agrobacterium*, and *Burkholderia* were isolated from more than one fungal host. Bacteria that showed highest sequence similarity with hitherto unknown, uncultured bacteria were isolated with both methods (**Figure 2**). A number of genera were only represented by bacterial strains isolated from one of the three fungal species with either the “liquid hyphal-baiting”- or “transfer-enrichment” method. With respect to bacterial preferences of adherence to different host fungi, both baiting methods recovered specialist as well as generalist antifungal bacteria (**Figure 3**).

### Potential Mycophagous Bacteria

**FIGURE 4:**
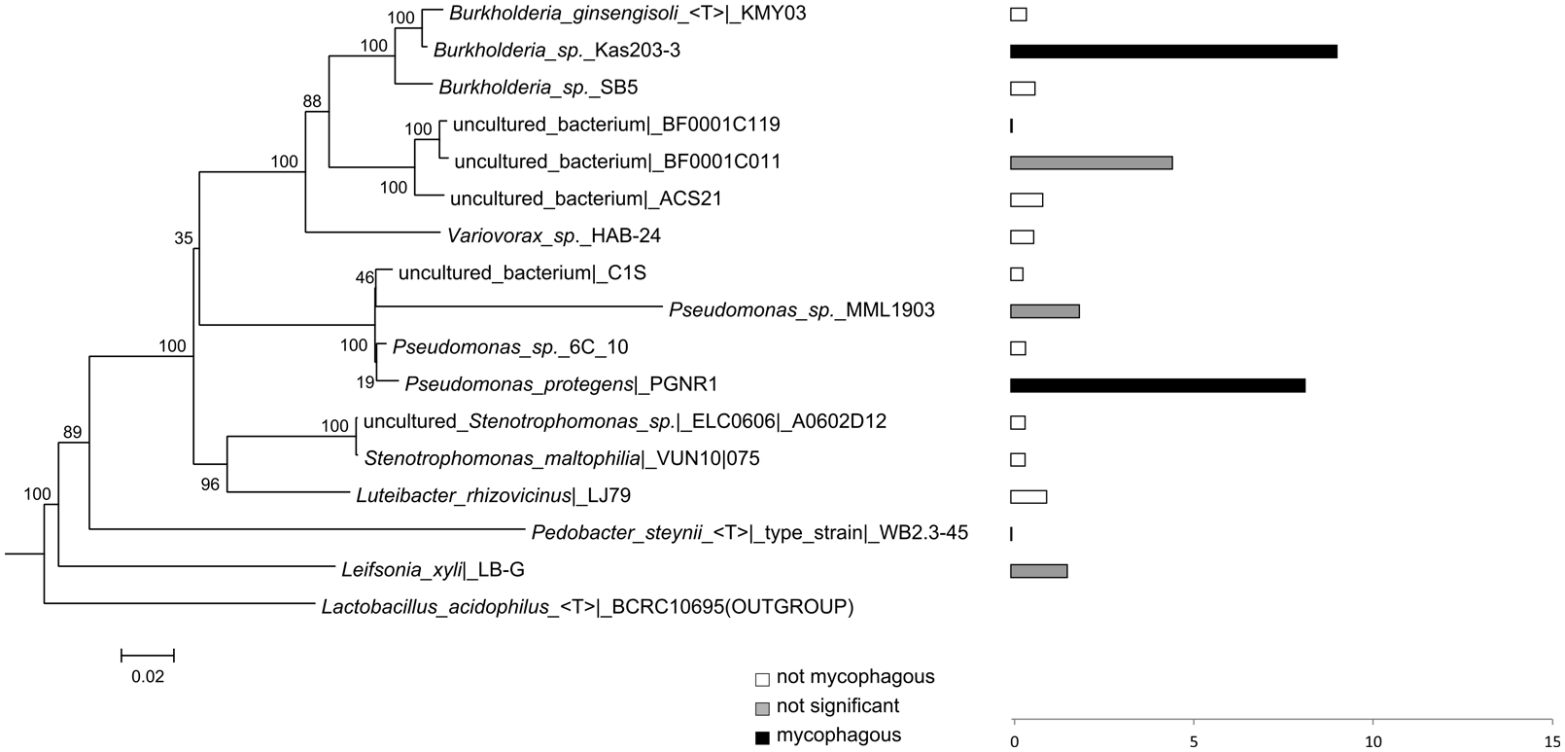
**Phylogeny and potential mycophagous ability of antifungal, hyphae-adhering bacteria retrieved with the short-term “liquid hyphal-baiting” approach, associated with *R. solani*.** The indicated bacterial strains are cultured or uncultured bacteria that have the closest match of 16S rDNA sequences to one or several of the antifungal, hyphae-adhering bacterial isolates. Bars indicate measured mycophagy ratios. Black bars show bacteria with significant mycophagous growth (ratio > 1; *P* < 0.05), grey bars represent bacteria with possible mycophagous growth (ratio > 1; *P* > 0.05), and white bars indicate bacteria with no mycophagous growth (ratio ≤ 1).

The percentage of antifungal bacteria that was able to grow on phytagel in the presence of their host fungus was overall highest for the “liquid hyphal-baiting” method. Here, 53% of the sequenced antifungal bacteria showed a positive growth response, compared to 33% of the sequenced antifungal isolates obtained by the “transfer-enrichment” method. In both methods, *M. hiemalis* was by far the most “attractive” fungus, stimulating growth of 80% (“liquid hyphal-baiting”) and 60% (“transfer-enrichment”) of all its antifungal colonizers, followed by *T. harzianum* (34.8 and 20%, respectively) and *R. solani* (12.5 and 33.3%, respectively; **Table 1**). Extensive colonization of fungal hyphae by mycophagous bacterial species could also be observed microscopically, sometimes inducing visible changes in hyphal morphology (See **Supplementary Figure S2** for examples). The actual number of antifungal bacteria with the ability to feed on fungi may have been higher, as for several isolates variation between replicates was considerable, resulting in a non-significant, positive mycophagy ratio (**Figures 3** and **4**).

**Table 1:** Occurrence of mycophagy among sequenced bacteria that adhered to fungal hyphae and were scored antifungal in the confrontation assay.

Fungus	Experiment	Reference	Total	Myc(%)	Not myc (%)	n.s.(%)	Myc ratio (avg)
*Trichoderma harzianum*	Liquid hyphal-baiting	Rudnick et al., 2015	23	34.8	30.4	34.8	5.4
*Mucor hiemalis*	Liquid hyphal-baiting	Rudnick et al., 2015	40	80	12.5	7.5	9.1
*Rhizoctonia solani*	Liquid hyphal-baiting	This study	16	12.5	68.8	18.8	8.6
**Overall**	**Liquid hyphal-baiting**	**This study**	**79**	**53.2**	**29.1**	**17.7**	**8.3**
*T. harzianum*	Transfer-enrichment	This study	10	20	40	40	7
*M. hiemalis*	Transfer-enrichment	This study	5	60	20	20	3.5
*R. solani*	Transfer-enrichment	This study	12	33.3	33.3	33.3	8.6
**Overall**	**Transfer-enrichment**	**This study**	**27**	**33.3**	**33.3**	**33.3**	**6.8**


*The strains are grouped by fungal host and isolation method. Given are the total number of strains (total), percentage of bacteria with significant mycophagous growth (myc), – with not significant mycophagous growth (n.s.) and – without mycophagous growth (not myc).*

While many potentially mycophagous isolates at the level of bacterial genera appeared to have a broad host range in terms of colonization, only a few strains could feed on more than one fungus (**Table 2**). With the “liquid hyphal-baiting” method, we isolated strains with closest match to *P. protegens* PGNR1 and *Burkholderia* sp. *Kas203-3* from all three fungi, but the bacteria did only significantly increase in biomass on *M. hiemalis* and *R. solani.* Other isolates, like strains with closest match to *Burkholderia* sp. SB5 and *Burkholderia phenazinium* Hg 10 only grew on *M. hiemalis* and *T. harzianum* whereas they were not found to colonize *R. solani.* Other examples of selective growth were strains assigned to the species *Burkholderia ginsengisoli* (*M. hiemalis)* and *Luteibacter rhizovicinus* (*M. hiemalis)* as well as a strain with closest match to an uncultured bacterium strain BF0001C119 from the genus *Oxalobacteraceae* (*T. harzianum*). Finally, some bacteria exhibited selective colonization but were not found to grow in the presence of the colonized fungi. Those were isolates closely related to *Pedobacter steynii* WB2.3-45 and the uncultured bacterium BF0001C011 (*Oxalobacteraceae)*. We also observed selective feeding for closely related “transfer-enrichment” isolates. Strains that had high similarity to *Pantoea allii* BD 390*, Pantoea ananatis* LMG 20105 and *Agrobacterium* sp. F72 colonized all three fungi but only grew in the presence of *R. solani* or *M. hiemalis* (see also **Table 2**).

**Table 2:** Mycophagous feeding patterns (mycophagy ratios per tested fungus) of antifungal bacterial isolates that were found to colonize hyphae of different several species.

Strain	*T. harzianum*	*M. hiemalis*	*R. solani*
**(A) Short-term liquid hyphal-baiting**			
*Pseudomonas protegens* PGNR1	0.6	14.8	8.2
*Burkholderia* sp. Kas203-3	1.2	16.4	9.1
*Burkholderia* sp. SB5	3	3.4	0.7
*Burkholderia phenazinium* Hg 10	3.6	3.8	
*Burkholderia ginsengisoli* (T) KMY03	0.9	3.2	0.4
*Luteibacter rhizovicinus* LJ79	n.s.(5,4)	2.2	1
Uncultured bacterium BF0001C119 *(Oxalobacteraceae)*	3.9		0
Uncultured bacterium BF0001C011 *(Oxalobacteraceae)*	n.s.(7,5)		n.s.(4,5)
*Pedobacter steynii* (T) WB2.3-45	0.9		0
**(B) Long-term “transfer-enrichment”**			
*Pantoea allii* (T) BD 390	1.2	0.8	7.7
*Pantoea ananatis* LMG 20105	1.2	2.9	0.8
Uncultured *Rahnella* sp. R707	5.4		n.s.(12,6)
*Agrobacterium* sp. F72	0.6	1.4	0.9


***(A)** short-term “liquid hyphal-baiting” and **(B)** long-term “transfer-enrichment”. Identification of the isolates is based on the closest match with the 16S rDNA sequences of cultured strains or environmental sequences of uncultured bacteria that are indicated in the table. Black background indicates high (7.7–16.4), gray intermediate (2.2–5.4), and white low (0–1.4) and non-significant mycophagy ratios (OD_600_ treatment/OD_600_ control), respectively. Missing data indicate that the bacterium was not isolated from the respective fungus, and therefore not tested; “n.s.” stands for “not significant” feeding because of variation between the replicates (average mycophagy ratios given in brackets). Data on strains obtained by “liquid hyphal-baiting” from hyphae of *M. hiemalis* and *T. harzianum* were retrieved from Rudnick et al. (2015; also see **Table 1**).*

### Validation of Mycophagous Growth

**FIGURE 5:**
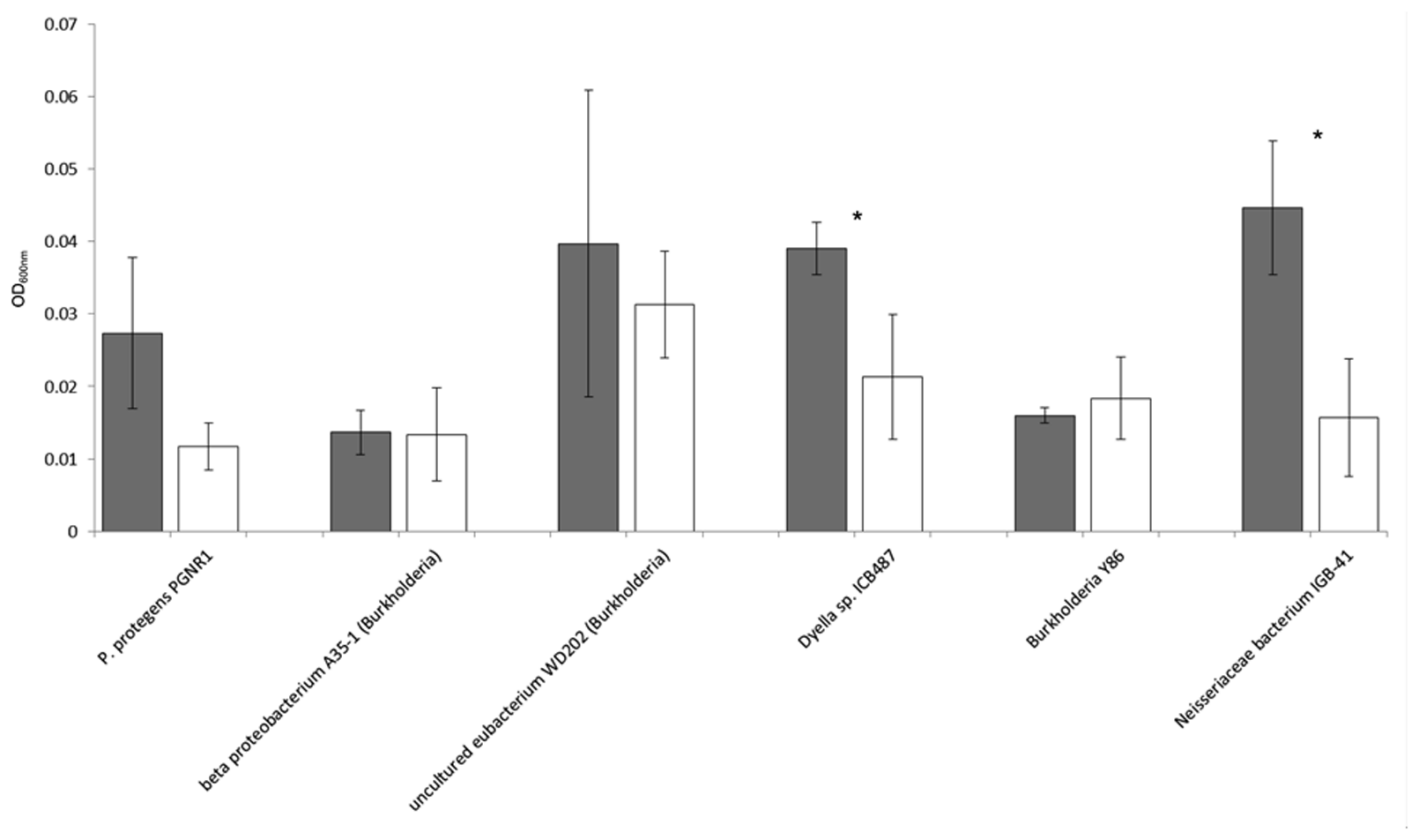
**Growth (OD_600nm_) of six potential mycophagous bacterial strains on liquid extracts obtained from intact fungal hyphae of the fungus *M. hiemalis* that had colonized Phytagel medium.** Gray bars indicate growth on the control (liquid extract of Phytagel only), white bars growth on hyphal extracts. Error bars show standard deviations and stars indicate significant differences between control and treatment, resulting from a two-tailed *t*-test.

With the six potentially mycophagous strains tested we did not obtain evidence that the fungus *M. hiemalis* exudes a sufficient amount of compounds on the Phytagel medium to sustain bacterial growth (**Figure 5**). On the contrary, a significantly higher biomass in liquid extracts from Phytagel only (control) as compared to liquid extracts from Phytagel colonized by fungi was observed for two bacterial strains (closest matches with *Neisseriaceae* bacterium IGB-41 and *Dyella* sp. ICB487, respectively).

## Discussion

The aim of this study was to compare two different methods to bait for rhizosphere bacteria with fungus-feeding abilities. Using short-term and long-term fungal baiting incubations, we isolated hyphae adhering bacteria from grass and sedge rhizospheric soils and evaluated the mycophagous potential of those bacteria that showed *in vitro* antifungal activity. The short-term baiting method (referred to as “liquid hyphal-baiting”) selected for quickly attaching bacteria of which several were able to use fungal compounds as the only source of energy. In the long-term “transfer-enrichment” approach, the most successfully fungus-feeding bacteria were transferred to a new microcosm, getting a head start in colonizing a “fresh” fungal host. Here, the bacteria that successfully competed with co-colonizers were enriched. The “liquid hyphal-baiting” method was already successfully used in a previous study (Rudnick et al., 2015).

### Fungal Inhibition

Since antifungal activity appears to be an important factor for mycophagous growth of *Collimonas* bacteria, all hyphae colonizing bacteria were screened for inhibition of the host fungus on a carbon-limited agar medium (WYA). Antifungal bacterial strains assigned to the genera *Rahnella, Pseudomonas, Pedobacter, Pantoea, Luteibacter, Leifsonia, Erwinia, Agrobacterium*, and *Burkholderia* were obtained from more than one fungal species. Notably, those generalists were also the most abundant ones, representing 80% of the antifungal, hyphae-adhering isolates. Species of the genera *Rahnella, Pseudomonas*, and *Burkholderia* have already been described as “universal fungiphile”, based on their ability to adhere to hyphae of a range of fungi (Warmink et al., 2009). Our study indicates that those bacteria are not only able to colonize a range of fungi but that they are also able to inhibit their growth. Specialist colonizers, i.e., bacterial species associated with only one fungus, were generally not very abundant.

### Isolation of Potential Mycophagous Bacteria

We successfully obtained potential mycophagous bacteria with both methods, yet, they yielded different sets of isolates (**Figure 3**). The “liquid hyphal-baiting” method selected for a diverse community of hyphae-attaching bacteria and a high number of fungus feeders. Especially *M. hiemalis* attracted a diverse community of antifungal bacteria of which 80% was potential mycophagous (average mycophagy ratio 9.1). At the genus level we observed overlaps in mycophagous isolates obtained with both methods (**Figure 2**).

The bacterial community, recovered with the “transfer-enrichment” method (**Figure 3**) was less diverse than the community recovered with the “liquid hyphal-baiting method” (**Figure 4** and Rudnick et al., 2015). This was probably due to the nature of the method: the repetitive stamping might have enriched only for the fastest and/or most competitively growing mycophagous bacteria. Only successful hyphal colonizers were enriched and transferred (“stamped”) to the next microcosm. Another possibility is that we enriched bacteria that were able to use compounds from Phytagel as a carbon source which would be more enriched in the transfer-method than in liquid-hyphal baiting, due to repetitive stamping.

### Phylogenetic Range of Potentially Mycophagous Bacteria Isolated

We isolated a broad range of potentially mycophagous bacteria with the two methods applied. Earlier studies used dilution plating on chitin yeast agar in combination with a rather laborious sand microcosm assay to isolate and demonstrate mycophagous growth of chitinolytic soil bacteria (De Boer et al., 2001). Baiting with growing fungi as the only source of carbon is a superior, more efficient method to obtain mycophagous bacteria. The baiting methods clearly indicated that mycophagy is not restricted to collimonads. In fact, we did not succeed to isolate collimonads with the “transfer-enrichment” method. Also the previous study only reported the isolation of one single *Collimonas* strain by the “liquid hyphal-baiting” method. The reason for our low success in isolating *Collimonas* bacteria might lie in the generally very low abundance of collimonads in rhizosphere bacterial communities (Hoppener-Ogawa et al., 2007). The two baiting methods allowed for the discovery of potentially mycophagous bacteria, belonging to different taxonomic groups (**Figure 2**). Our study also showed that the class *Burkholderiales* which is known to be engaged in interactions with eukaryotic hosts (Stopnisek et al., 2015) harbors many other mycophagous bacteria besides the genus *Collimonas.* It is remarkable that none of the fungus associated isolates belonged to the bacterial phylum of *Firmicutes*. *Bacilli* and especially *Paenibacilli* are prominent genera of the *Firmicutes* which have been reported to be able to (internally and externally) colonize hyphae of plant pathogenic fungi as well as beneficial ectomycorrhizal fungi and fungal plant endophytes (Dijksterhuis et al., 1999; Toljander et al., 2006; Hoffman and Arnold, 2010). The absence of *Firmicutes* in our study may be due to the fact that the appropriate fungal hosts were not included. Previous studies revealed that fungal identity can influence hyphae-adhering mycophagous bacteria.

### Non-mycophagous Bacteria

The ability to metabolize fungus-derived carbon as the only source of energy varied among the “generalist” hyphal colonizers. A range of bacteria seemed to be able to attach to fungal hyphae. However, for several bacterial isolates colonization of a fungal host appeared not to be associated with the ability to feed on it, despite the fact that they had inhibitory activity against the host fungus in *in vitro* screenings. This could mean that those bacteria lack the specific molecular machinery to attack the fungus and are thereby restricted to feed only on energy resources that spontaneously leak out of the fungus. If energy resources are leaking out of the fungus as result of the activities of mycophagous bacteria, the growth of non-mycophagous attaching bacteria could be considered as cheaters (profit without investment; Hibbing et al., 2010). It could also be possible that some mycophagous bacteria are only able to feed on fungi when being part of a multi-species consortium of hyphal colonizers, concerting the release of antifungal substances. Those bacteria would be scored as “non-mycophagous” in our assay.

### Confirmation of Mycophagy

According to the definition given by Leveau and Preston (2008) mycophagous bacteria should be actively involved in getting access to fungal nutrients, e.g., by causing leakage of fungal membranes. The mycophagy test on Phytagel could also indicate a positive response of isolates that merely grow on fungal exudates without any impact of the bacteria on the eﬄux of fungal nutrients. To prove that bacterial isolates are real mycophagous, detailed studies, including microscopic observation and determination of growth responses of mutants, are needed for each bacterial–fungus combination. Still, we think that our assay is pointing at real mycophagous bacteria. Our arguments for this are as follows: (1) we focused only on bacteria that showed antifungal properties under nutrient-poor conditions (WYA). Under these conditions, many compounds with antifungal activity can disturb the cell membrane integrity and cause leakage (Ghannoum and Rice, 1999). (2) Isolated hyphae-adhering bacteria showed a specific growth response or no response at all when encountering hyphae of the three fungal species on Phytagel, making it less likely that the passive eﬄux of fungal exudates on this nutrient-poor medium can explain bacterial growth. (3) In fact, when confronting six potentially mycophagous bacterial strains with extracts of Phytagel that had previously been colonized by hyphae of the fungus *M. hiemalis*, we did not observe growth (**Figure 5**). On the contrary, for two strains (assigned to *Neisseriaceae* bacterium IGB-41 and *Dyella* sp. ICB487, respectively) we even detected significantly less bacterial growth on fungal exudates as compared to the control. This suggests that the amount of passively released exudates by fungi on the nutrient poor Phytagel medium is not enough to provide nutrients for bacterial growth. Elevated growth on the control extracts could be caused by the fungal withdrawal of nutrients from the Phytagel. Thus, we think that the most plausible explanation for the observed results is bacterial growth on fungal hyphae. Such bacteria have to be actively engaged in the acquisition of fungal nutrients and can therefore be considered as mycophagous bacteria.

### Potential Plant Pathogens

With the “liquid hyphal-baiting” method, it has already been shown that several bacterial colonizers of *M. hiemalis* and *T. harzianum* belong to genera that harbor plant-pathogenic bacteria (*Curtobacterium, Enterobacter*, and *Leifsonia*; e.g., Rudnick et al., 2015). Using the “transfer-enrichment” method, we found more bacteria belonging to such genera (*Pantoea, Agrobacterium*, and *Erwinia*, e.g.). The repeated isolation of potential plant-pathogenic bacteria with different baiting methods may indicate that the fungal hyphae provide plant-pathogenic bacteria with a good habitat. The benefit of associating with an alternate eukaryotic host is unclear. Since many saprotrophic and pathogenic fungi are able to colonize plant roots endophytically, the colonization of fungal hyphae in combination with movement along the “fungal highway” (Kohlmeier et al., 2005) might enable those potential plant pathogens to infect plant roots using the fungus as a vector. This is known for other host–bacterium interactions. The *Vibrio cholera* infection process is for example facilitated by the colonization of a protozoan vector which is subsequently taken up by the human host through contaminated drinking water (Nelson et al., 2009). Potential plant pathogens could also benefit from colonizing and feeding on alternate eukaryotic hosts like fungi under circumstances when their preferred plant host is not available. This could for example be the case for seasonal crop plants that only represent a favorable host during growing season. Once such crops get decomposed in autumn, the fungal community would serve as refuge until the next growing season begins.

### Summary

We present an innovative transfer-enrichment approach to enrich antifungal, mycophagous bacteria from soil and compared it to another short-term baiting method. Both methods retrieved distinct and phylogenetically diverse sets of inhibitory, mycophagous rhizosphere bacteria. Our results give more support to the previously indicated potential of many rhizosphere bacteria to grow on fungal resources. The comparison of the two baiting methods might form a basis for future screenings and applications of associations between mycophagous bacteria and fungi.

## Conflict of Interest Statement

The authors declare that the research was conducted in the absence of any commercial or financial relationships that could be construed as a potential conflict of interest.

## Supplementary Material

**FIGURE S1 | Experimental setup of the confrontation assay.** During colony expansion, the confrontation of the fungus (center) and bacterial colonies (blue) is forced.

**FIGURE S2 | Microscopic images of the mycophagy assay.** Growth of bacterial strains *Burkholderia* sp. Kas203-3 and *Luteibacter rhizovicinus* on the fungi *Mucor hiemalis, Trichoderma harzianum*, and *Rhizoctonia solani* is shown. Controls showing bacteria or fungi inoculated on Phytagel medium alone are presented, as well.
