# Supplementary figures and images for: Methods for Baiting and Enriching Fungus-Feeding (Mycophagous) Rhizosphere Bacteria
Source: Front Microbiol. 2015 Dec 22;6:1416. doi: 10.3389/fmicb.2015.01416 (PMC4687392; doi:10.3389/fmicb.2015.01416)

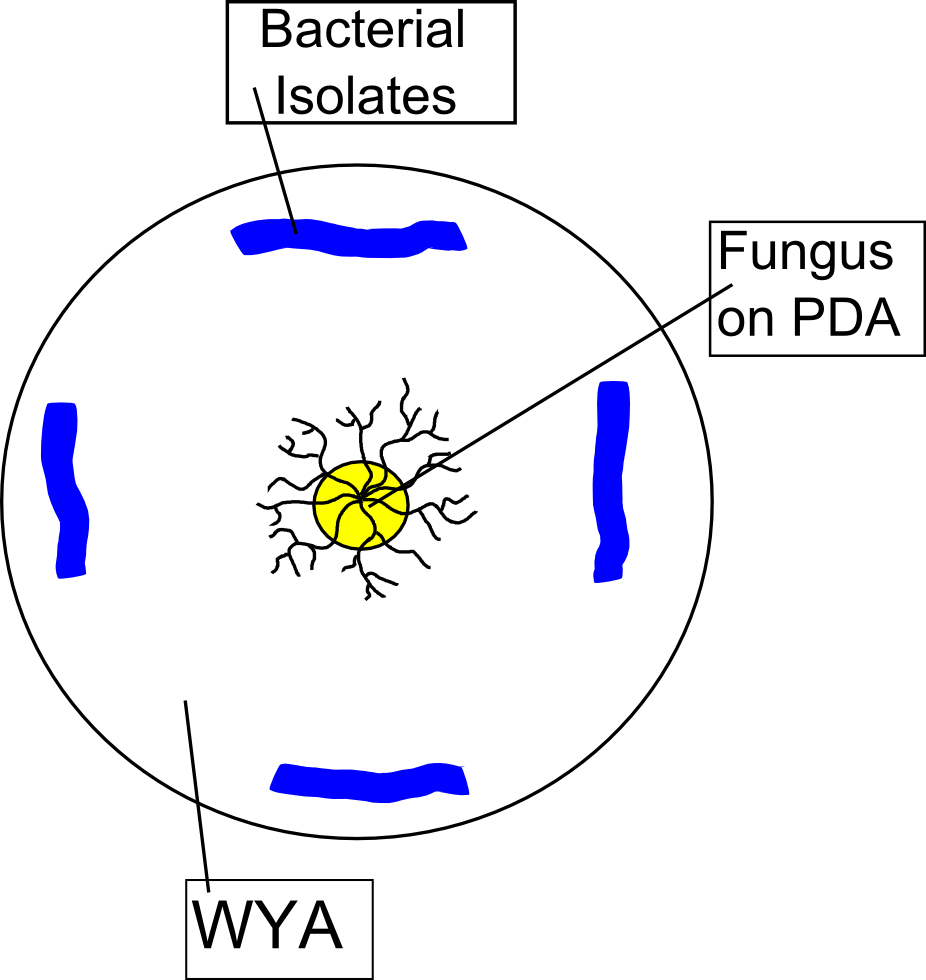

Supplement: Supplementary file 1 [file Image_1.TIFF]

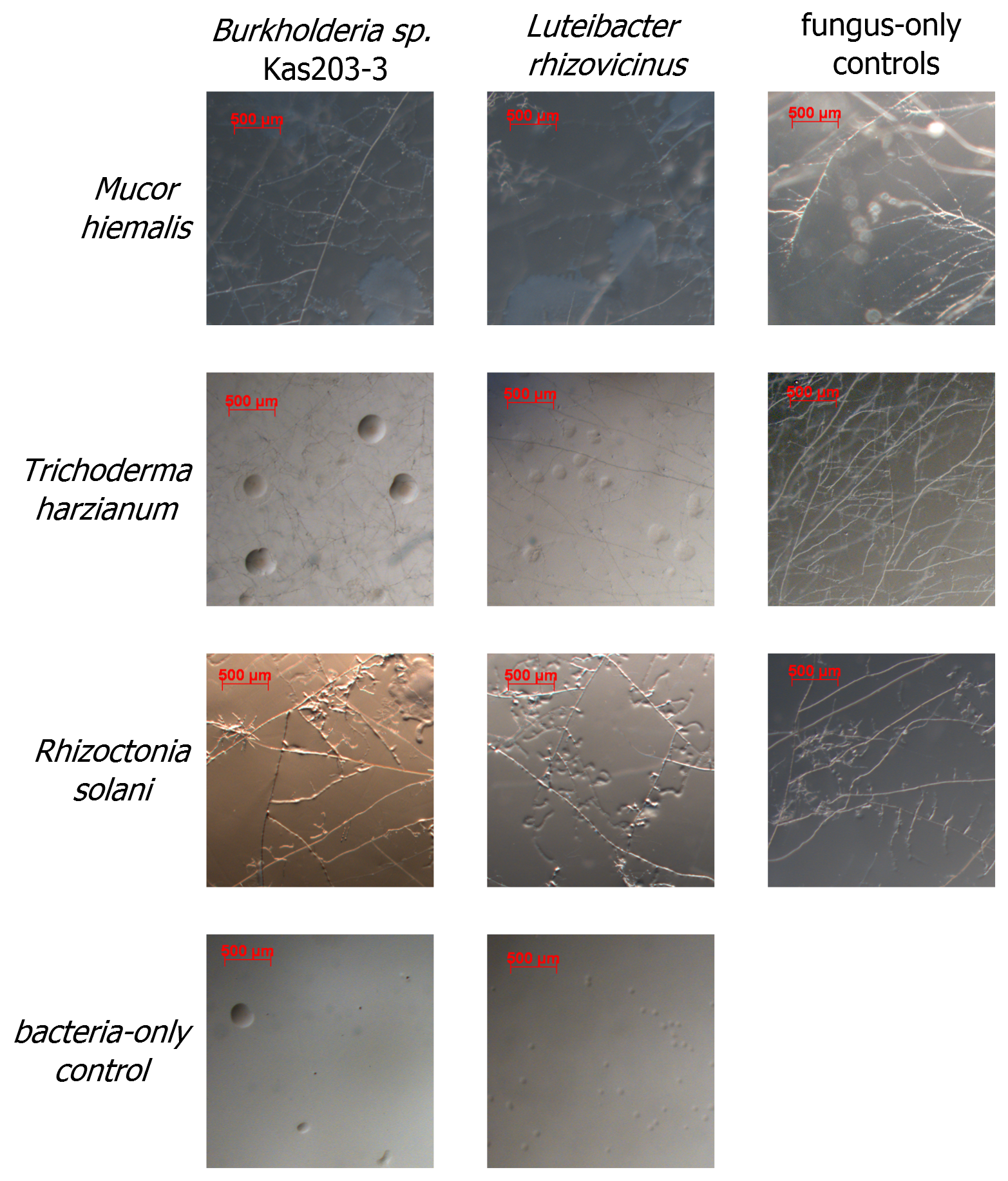

Supplement: Supplementary file 2 [file Image_2.TIFF]
